# Type 2C Phosphatase 1 of *Artemisia annua* L. Is a Negative Regulator of ABA Signaling

**DOI:** 10.1155/2014/521794

**Published:** 2014-10-28

**Authors:** Fangyuan Zhang, Xueqing Fu, Zongyou Lv, Qian Shen, Tingxian Yan, Weiming Jiang, Guofeng Wang, Xiaofen Sun, Kexuan Tang

**Affiliations:** Plant Biotechnology Research Center, Fudan-SJTU-Nottingham Plant Biotechnology R&D Center, School of Agriculture and Biology, Shanghai Jiao Tong University, 800 Dongchuan Road, Shanghai 200240, China

## Abstract

The phytohormone abscisic acid (ABA) plays an important role in plant development and environmental stress response. Additionally, ABA also regulates secondary metabolism such as artemisinin in the medicinal plant *Artemisia annua* L. Although an earlier study showed that ABA receptor, AaPYL9, plays a positive role in ABA-induced artemisinin content improvement, many components in the ABA signaling pathway remain to be elucidated in *Artemisia annua* L. To get insight of the function of AaPYL9, we isolated and characterized an AaPYL9-interacting partner, AaPP2C1. The coding sequence of AaPP2C1 encodes a deduced protein of 464 amino acids, with all the features of plant type clade A PP2C. Transcriptional analysis showed that the expression level of AaPP2C1 is increased after ABA, salt, and drought treatments. Yeast two-hybrid and bimolecular fluorescence complementation assays (BiFC) showed that AaPYL9 interacted with AaPP2C1. The P89S, H116A substitution in AaPYL9 as well as G199D substitution or deletion of the third phosphorylation site-like motif in AaPP2C1 abolished this interaction. Furthermore, constitutive expression of AaPP2C1 conferred ABA insensitivity compared with the wild type. In summary, our data reveals that AaPP2C1 is an AaPYL9-interacting partner and involved in the negative modulation of the ABA signaling pathway in *A. annua* L.

## 1. Introduction

The phytohormone abscisic acid (ABA) is a key regulator of plant developmental processes and plant responses to abiotic stresses including drought, salt, osmotic, and cold stress [1, 2]. ABA acts through a complex signaling cascade to induce changes in gene expression and in adaptive physiological responses [3]. In 2009, members of the PYR1/PYL/RCAR family of proteins (hereafter referred to as PYLs for simplicity) are proved to be ABA receptors in the cytoplasm and nucleus [4]. Upon ABA binding, the PYLs interact with and inhibit most members of the clade A subfamily of type-2C protein phosphatases (PP2Cs) [5]. ABA-mediated PP2C inhibition leads to the activation of subclass III SnRK2s [6]. Once activated, SnRK2s phosphorylate the downstream transcription factors and slow sustained (S-type) anion channels [7]. In this model, PP2C serves as a central and negatively regulated hub in ABA signaling [8] and the first connection of the ABA signaling with reversible phosphorylation.

Though plant PP2C is encoded by multigene family with 80 and 78 members in* Arabidopsis* and rice, respectively [9], only clade A subfamily is considered to be involved in ABA signaling [10]. Currently, at least six PP2Cs belonging to clade A are known to negatively regulate ABA signaling, namely, ABI1, ABI2, PP2CA/AHG3, AHG1, HAB1, and HAB2 [11]. Many of these protein phosphatases are induced by ABA. Two* Arabidopsis* strong ABA-insensitive* loci*, ABI1 and ABI2, have been demonstrated to encode PP2C enzymes, which functionally inhibit SnRK2s autophosphorylation [12]. Studies on the knockout and double mutants in ABI1 and ABI2 confirmed that ABI1 and ABI2 have partially redundant function as inhibitor of ABA signaling [13].

In addition to* Arabidopsis*, many PP2Cs have been studied in various plant species [14]. In rice, three PP2Cs were demonstrated interaction with OsPYR/RCAR5 in ABA dependent manner [15]. When screening PP2Cs involved in beechnut dormancy, FsPP2C1 is identified and characterized as a negative regulator of ABA signaling in seed dormancy [16]. Subsequently, FsPP2C1 is proved interaction with* Arabidopsis* ABA receptor PYL8 in nucleus [17]. Overexpression of* PP2C* gene from maize decreased plant tolerance to drought and salt in* Arabidopsis* [18]. In moss* Physcomitrella patens*, genetic evidence demonstrated that two PP2Cs functionally acted as a negative regulator in ABA signaling, which indicate that ABA signaling is evolutionarily conserved between* Arabidopsis* and* P. patens* [19]. In addition to negative regulator in ABA responses, a PP2C from strawberry was reported to be a negative regulator in fruit ripening process [14].

Malaria is a global health problem especially in torrid zone, with more than one billion people living in areas with a high risk of the disease [20]. Artemisinin, isolated from traditional Chinese herb* Artemisia annua* L. (Qing Hao), is a sesquiterpene lactone endoperoxide that provides the basis for effective treatments of malaria especially for the cerebral and the chloroquine-resistant forms of this disease [21]. Besides the antimalarial activity, artemisinin has also been reported to antiviral [22], anticancer [23], and antischistosomal activities [24]. We previously reported that the content of artemisinin is induced by exogenous ABA [25] and overexpression of AaPYL9, a functional ABA receptor in* Artemisia* [26].

To get deeper insight into the ABA signaling in* Artemisia* and ABA-regulated secondary metabolism, we started a search for putative PP2C which interacts with ABA receptor AaPYL9. A cDNA library was constructed from* Artemisia* leaves and sequenced. Bioinformatics analysis identified that a PP2C belongs to clade A PP2C subfamily, which is named AaPP2C1. AaPP2C1 interacts with AaPYL9 in yeast two-hybrid and BiFC assays. Moreover, overexpression of AaPP2C1 in* Arabidopsis* produces insensitivity to ABA in seed germination and root elongation. Taken together, our data reveal that AaPP2C1 is an AaPYL9-interaction partner and play a negative regulator role in ABA signaling.

## 2. Materials and Methods

### 2.1. Chemicals, Plant Materials, Growth Conditions, and Stress Treatments

Abscisic acid was obtained from Sigma-Aldrich (http://www.sigmaaldrich.com);* Artemisia annua* L. used in this study was the same as previously described which were grown in a controlled environment with 16/8-h light/dark photoperiod at 26°C [26]. For abiotic stresses and ABA treatment, 1-month-old* A. annua* were treated with 300 mM NaCl and 10 *μ*M ABA, followed by sampling at 0, 3, 6, and 12 h. Drought stress was performed by leaving the intact 1-month-old* A. annua* in the air without water supply, followed by sampling at 0, 3, 6, and 12 h.

The* Arabidopsis* (*Arabidopsis thaliana*) were grown in pots in a growth chamber under 24°C and 16-h-light/8-h-dark photoperiod at 80 to 100 mE m-2 s-2. To examine the expression of ABA-responsive genes, wild type and AaPP2C1-overexpression plants grown in liquid medium were treated with 10 *μ*M ABA for 3 h, and the expression levels of* RD29A*,* RD29B*,* P5CS1,* and* RAB18* were determined. For statistical analysis, at least three independent experiments were performed. *P* values were calculated using the Student's *t*-test.

### 2.2. Mutagenesis of AaPYL9

The point mutation of* AaPYL9* used in this study was described previously [26]. The mutation of* AaPP2C1* was performed using overlapping PCR strategy as work in AaPYL9.

### 2.3. Plasmid Construction

To overexpression of AaPP2C1 in* Arabidopsis*, the coding sequence of AaPP2C1 was amplified using the following primers: AaPP2C1-F-Bgl: (5′-GAAGATCTATGGAAGATATCCCTCCTTC-3′) and AaPP2C1-R-Sac: (5′-GCGTCGACTCAAGATTTAGTTTTAAACC-3′). After being confirmed by sequencing, the fragment was cloned into the* BamH*I and* Sac*I sites of binary vector pHB under the control of the double CaMV 35S promoter.

For yeast two-hybrid assay, the full length CDS as well as series of deletion and point mutation of AaPP2C1 were cloned into pGADT7 vector between* BamH*I and* Sal*I sites with the following primers: AaPP2C1-F-Bgl and AaPP2C1-R-Sal (5′-GCGTCGACTCAAGATTTAGTTTTAAACC-3′). The full length CDS and point mutation of AaPYL9 were amplified by the following primers: AaPYL9-F-EcoR (5′-CGGAATTCATGAAGTACAGTAAGAAAGT-3′) and AaPYL9-R-BamH (5′-CGGGATCCTCACTGAGTAATGTTCAGC-3′), the PCR fragments inserted into* EcoR*I and* BamH*I sites of pGBKT7 vector subsequently.

For subcellular localization of AaPP2C1, the CDS of AaPP2C1 was amplified by PCR and cloned into gateway cloning vector pENTR (Invitrogen), then subsequently transferred to the destination vector pEarleyGate104 [27] by Gateway LR recombination reaction (Invitrogen), thus generating pEarleyGate104-YFP-AaPP2C1 construct. The pEarleyGate104-YFP-AaPYL9 construct used in this study was described previously [26].

BiFC vectors pEarleyagte201-YN and pEarleygate202-YC were kindly provided by Professor Steven J. Rothstein [28]. pENTR-AaPYL9 was transferred into pEarleyagte202YC and pENTR-AaPP2C1 was transferred into pEarleyagte201YN by Gateway LR recombination reaction (Invitrogen), named pEG202YC-AaPYL9 and pEG201YN-AaPP2C1, respectively.

### 2.4. Construction of AaPP2C1 Transgenic* Arabidopsis*

The pHB-AaPP2C1 construct was introduced into* Agrobacterium tumefaciens* strain GV3101 and then infiltrated into* Arabidopsis* with floral-dip method. The transgenic seedlings were selected in hygromycin medium (25 *μ*g mL^−1^). T2 plants that produced 100% hygromycin-resistant plants in T3 generation were considered homozygous and used for further studies.

### 2.5. Real-Time Quantitative PCR Analysis

The real-time quantitative PCR analysis was performed as previously described [26]. Briefly, total RNA was extracted from roots, stem, leaves, flower buds, flowers, and samples after treatment. The relative expression levels were normalized by the housekeeping gene* ACTIN*. To examine the expression of ABA-responsive genes, total RNA was isolated from wild type and AaPP2C1-overexpression* Arabidopsis* as described above. The primers used in this research were described in Supplementary Table 1 (see Supplementary Material available online at http://dx.doi.org/10.1155/2014/521794).

### 2.6. Yeast Two-Hybrid Assay

Sets of bait and prey constructs were cotransformed into AH109 yeast strain. The transformed yeast cells were selected on synthetic minimal double dropout medium deficient in Trp and Leu. Protein interaction tests were assessed on triple dropout medium deficient in Trp, Leu, and His or quadruple dropout medium deficient in Trp, Leu, His, and adenine. At least six clones were analyzed, and experiments were repeated three times with similar results.

### 2.7. Subcellular Localization and BiFC Analysis

The subcellular localization analysis was performed as previously described [26]. BiFC assays were conducted according to Lu et al. [28]. Briefly, the constructs of AaPP2C1-cYFP, AaPP2C1m1-cYFP, and AaPP2C1m2-cYFP were introduced into* A. tumefaciens* strain GV3101 as well as AaPYL1-nYFP, AaPYL9m1-nYFP, and AaPYL9m2-nYFP constructs. Pairs of combinations were coinfiltrated into* N*.* benthamiana*. YFP signal was observed 48 to 60 h after being infiltrated by confocal laser scanning microscope (Leica TCS SP5-II). Results were confirmed by at least three times repeats.

### 2.8. Germination and Root Growth Assay

The germination and root growth assay were performed as previously described [26]. Briefly, the wild type and transgenic* Arabidopsis* seeds were plated on MS medium with or without different concentrations of ABA (0.1 *μ*M and 0.3 *μ*M) after being stratified for 3 d at 4°C. Seedling establishment was scored as the percentage of seeds that developed green expanded cotyledons and the first pair of true leaves at 7 d. Root growth was measured after 7 d of transferring of 5-day-old seedlings onto vertical MS plates containing 5 *μ*M ABA for ABA sensitivity assay.

## 3. Results and Discussion

### 3.1. Isolation and Sequence Analysis of AaPP2C1 in* A. annua*

Recently, we cloned and characterized AaPYL9, an ABA receptor orthologue in* A. annua*, which plays an important role in regulating artemisinin biosynthesis [26]. To identify the partners and downstream targets of AaPYL9, we performed bioinformatic analysis of cDNA library by BLAST-P program. A candidate gene which encodes a PP2C protein and contains the typical PYLs interaction domain was selected for further study. The cDNA fragment of this gene was isolated from* A. annua via* RT-PCR and designated as* AaPP2C1* which was 1392 bp long and encodes 463 amino acids. The deduced AaPP2C1 protein had a calculated molecular mass of 50.2 kDa and a predicted pI of 4.92 (http://web.expasy.org/compute_pi/).

**Figure 1:**
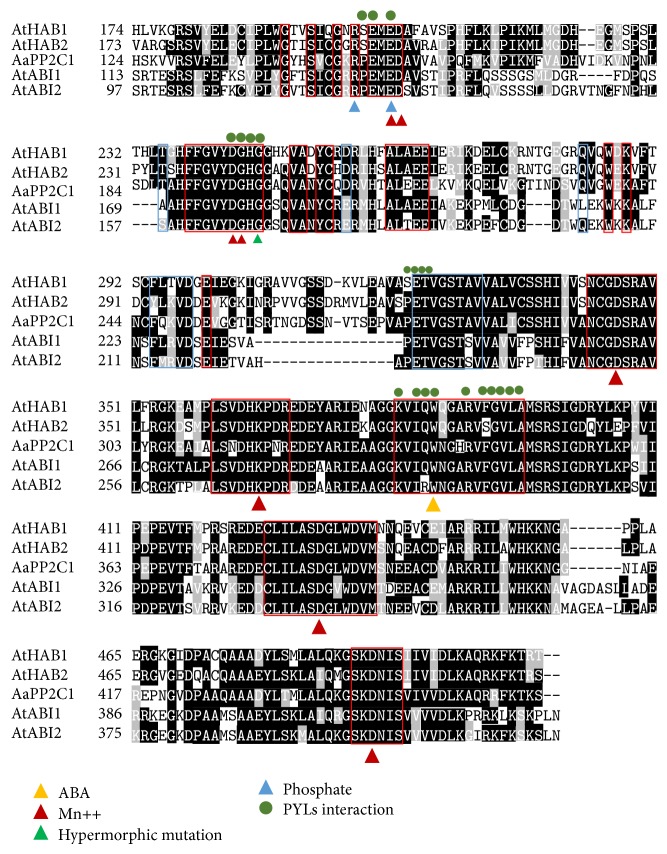
Alignment of AaPP2C1 with four* Arabidopsis* PP2Cs. Amino acid sequence alignment of* Artemisia* AaPP2C1 with four* Arabidopsis* PP2Cs. Red frame indicates the amino acid residues involved in the interaction with ABA receptors; blue frame indicates the amino acid residues involved in the phosphorylation. The different icon indicates contact points with phosphate, metal, ABA, hypermorphic mutations and two mutation sites produced in this study.

The phylogenetic relationship between AaPP2C1 and 68* Arabidopsis* PP2Cs was shown in Supplementary Figure 1. Phylogenetic analysis indicated that AaPP2C1 is clustered with group A PP2Cs of* Arabidopsis*. Sequence alignment with ABI1, ABI2, HAB1, and HABI2 from* Arabidopsis* reveals that AaPP2C1 had the same motif with them, such as PA- (phosphatidic acid-) binding site, PYLs interaction site, and a conserved PP2C domain (Figure 1).

### 3.2. *AaPP2C1* Mainly Expressed in Leaves and Is Induced by ABA Treatment and Abiotic Stress

**Figure 2:**
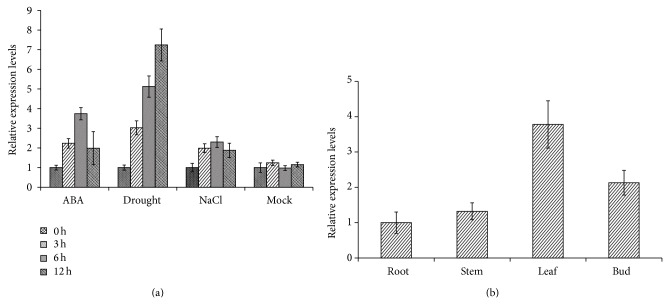
Expression profiling analysis of* AaPP2C1*. (a) qRT-PCR analysis of the expression pattern of* AaPP2C1* under various environmental stress conditions. (b)* AaPP2C1* expression in various tissues. The data represent the means ± SD (standard deviation) of three repeated samples.

Expression pattern analysis can provide important clues for biological function. Quantitative RT-PCR was used to examine the expression pattern of* AaPP2C1* gene in different tissue and under different treatments. As shown in Figure 2, the transcripts of* AaPP2C1* could be detected in whole plants including root, stem, leaves, and flowers, while they highly accumulates in leaves; this is in accord with the expression pattern of* AaPYL9* [26]. On account of many identified ABA signalling component genes were induced under stress condition and ABA treatment; the expression level of* AaPP2C1* under drought stress, ABA, and NaCl solution treatment were determined by quantitative real-time PCR as well. The results showed that the expression of* AaPP2C1* was significantly induced under either drought stress or ABA treatment or NaCl treatment. The similar expression pattern in different tissue between* AaPP2C1* and* AaPYL9* suggests that AaPP2C1 might interact with AaPYL9. The inducible transcript of* AaPP2C1* suggested that this gene participates in ABA or drought responses.

### 3.3. Subcellular Localization of AaPP2C1

**Figure 3:**
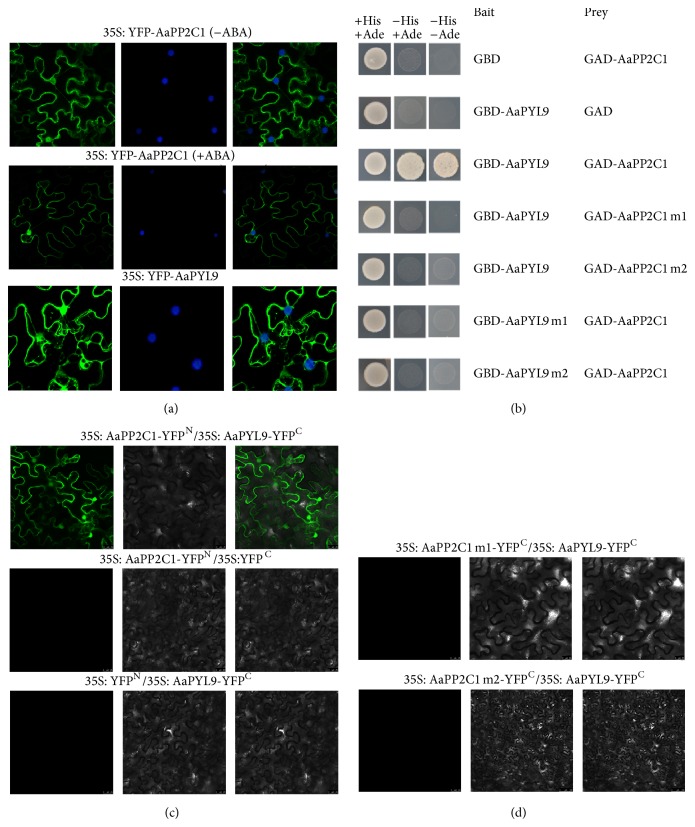
AaPP2C1 localizes at both cytosol and nucleus and interaction with AaPYL9. (a) Subcellular localization of AaPP2C1 and AaPYL9 in* Agrobacterium*-infiltrated tobacco leaves. Nucleus was visualized by DAPI staining. Left panel: YFP channel; middle panel: DAPI channel; right panel: merged picture. Treatment with 10 *μ*M ABA for 1 h did not change the subcellular localization of AaPP2C1. (b) The construct of genes fused N- and C-terminal to YFP, respectively (as indicated), were coinfiltrated into tobacco leaves. YFP signal was observed 48 h to 60 h after infiltrated. (c) The BiFC assay between two mutations of AaPP2C1 and AaPYL9. (d) Yeast two-hybrid assay confirming the interaction between AaPP2C1 and AaPYL9. Bait indicates the protein fusion with Gal4 BD domain. Prey indicates the protein fusion with Gal4 AD domain. GBD is empty pGBKT7 vector, GAD is empty pGADT7 vector. (+His, +Ade) indicates Leu-Trp SD medium; (−His, +Ade) indicates Leu-Trp-His SD medium; (−His, −Ade) indicates Leu-Trp-His-Adedeficient SD medium. AaPYL9m1: substitution mutation P89S; AaPYL9m2: substitution mutation H116A. AaPP2C1m1: substitution mutation G199D; AaPP2C1m2: deletion mutation (four amino acid residues from 305 to 308: RGKE).

To determine the subcellular localization of AaPP2C1 in plant cells, we performed* in vivo* targeting experiments in tobacco (*Nicotiana benthamiana*). Both 35S: YFP-AaPP2C1 and 35S: YFP-AaPYL9 constructs were delivered into epidermis leaf cell of tobacco by* A*.* tumefaciens *infiltration. Consistent with subcellular localization in* Arabidopsis* protoplasts as previously reported [26], YFP-AaPYL9 fluorescence was observed in both the nucleus and the cytosol (Figure 3(a)). Similar to YFP-AaPYL9, AaPP2C1-YFP fluorescence signal was observed in nucleus and cytosol. In addition, ABA treatment did not modify the subcellular localization of AaPP2C1-YFP and AaPYL9-YFP under our experimental conditions (Figure 3(a)).

### 3.4. AaPP2C1 Interact with AaPYL9 in Yeast Two-Hybrid Assay

We previously reported that AaPYL9 interacted with ABI1 but not ABI2, HAB1, or HAB2 from* Arabidopsis* in yeast two-hybrid assay and* in planta*. To test whether the AaPP2C could interact with AaPYL9, yeast two-hybrid assay was performed between AaPP2C1 and AaPYL9. The full length of AaPP2C1 was fused to the GAL4 activation domain (GAD) while AaPYL9 was fused to the GAL4 DNA binding domain (GBD), generating AD-AaPP2C1 and BD-AaPYL9 constructs, respectively. Yeast cells cotransformed with AD-AaPP2C1 and BD-AaPYL9 grew well on selective medium, whereas the combination of BD-AaPYL9 with empty pGADT7 did not activate transcription of the* HIS3* and* ADE2* reporter gene (Figure 3(b)). This result indicated that AaPP2C1 could interact with AaPYL9 in yeast two-hybrid assay.

Recent structural and genetic researches revealed that a gate-latch-lock mechanism in ABA receptors physically interacts with PP2Cs [4, 29]. To address the question of whether AaPP2C1 might function in ABA signaling through physical interaction with AaPYL9 underlying the gate-latch-lock mechanism, two mutated AaPYL9s were generated, AaPYL9m1 (substitution mutation P89S which is located on gate region) and AaPYL9m2 (substitution mutation H116A which is located on latch region), then subsequently fused to GBD. In combination with the prey construct AD-AaPP2C1, the GBD-AaPYL1m1 as well as GBD-AaPYL9m2 did not activate the reporter genes transcription (Figure 3(b)). These results showed that AaPP2C1 interact with AaPYL9 underlying gate-latch-lock mechanism, and the mutations on gate or latch region can disrupt the AaPP2C1-AaPYL9 interaction.

Eukaryotic PP2Cs containing a converse catalytic domain at either the N- or the C-terminus [30]. The metal coordinating centre in the catalytic domain is critical for their phosphatase activity and physiological function in ABA signaling. The dominant ABA-insensitive ABI1G180D (encoded by* abi1*-*1*) and ABI2G168D (encoded by* abi2*-*1*) disrupt the interaction between the PP2Cs and the ABA receptors PYR/PYL/RCAR in* Arabidopsis* [4, 29]. In addition, a CDPK phosphorylation site-like motif (CPL) in ABI2 is required for the interactions of ABI2 with PYL5 and PYL9 [31]. To investigate the important site of AaPP2C1 involved in interaction with AaPYL9, a point substitution mutation located on catalytic domain (AaPP2C1m1) and a deletion mutation (four amino acid residues from 305 to 308: RGKE) of AaPP2C1 (AaPP2C1m2) were generated and subsequently fused to GAD. In the case of yeast cells cotransformed with GAD-AaPP2C1m1 and GBD-AaPYL9 as well as the combination of AD-AaPP2C1 m2 with BD-AaPYL9 did not activate transcription of the* HIS3* and* ADE2* reporter gene (Figure 3(b)). These results indicate that the two AaPP2C1 mutations, AaPP2C1m1 and AaPP2C1m2, abolished the interaction with AaPYL9 in yeast two-hybrid; functional PP2C catalytic domain and the third phosphorylation site-like motif of AaPP2C1 are required for the interaction of AaPP2C1 and AaPYL9.

### 3.5. *In Planta* Interaction between AaPP2C1 and AaPYL9

Bimolecular fluorescence complementation (BiFC) assays were used to detect the interaction between AaPP2C1 and AaPYL9 in plant cell. To this end, AaPP2C1 was fused to the N-terminal (amino acid 1–174) of YFP protein (YFP^N^) in the pEG201YN vector, which generated an AaPP2C1-YFP^N^ protein. AaPYL9 was fused to the C-terminal (amino acid 175–239) of YFP protein (YFP^C^) in the pEG202YC vector, which generated an AaPYL9-YFP^C^ protein. The corresponding constructs were coinfiltrated into leaf cells of tobacco; YFP fluorescence signal was observed in both of nucleus and cytosol (Figure 3(c)). As control, no fluorescence signal was observed when empty pEG201YN vector was coinfiltrated with pEG202YC-AaPYL9 or when pEG201YN-AaPP2C1 was coinfiltrated with empty pEG202YC vector (Figure 3(c)). Moreover, in accord with previous finding in the yeast two-hybrid assay, the AaPP2C1m1 and AaPP2C1m2 cannot interaction with AaPYL9 in the BiFC assay (Figure 3(d)).

### 3.6. Overexpression of* AaPP2C1* Showed a Reduced Sensitivity to ABA in* Arabidopsis*

**Figure 4:**
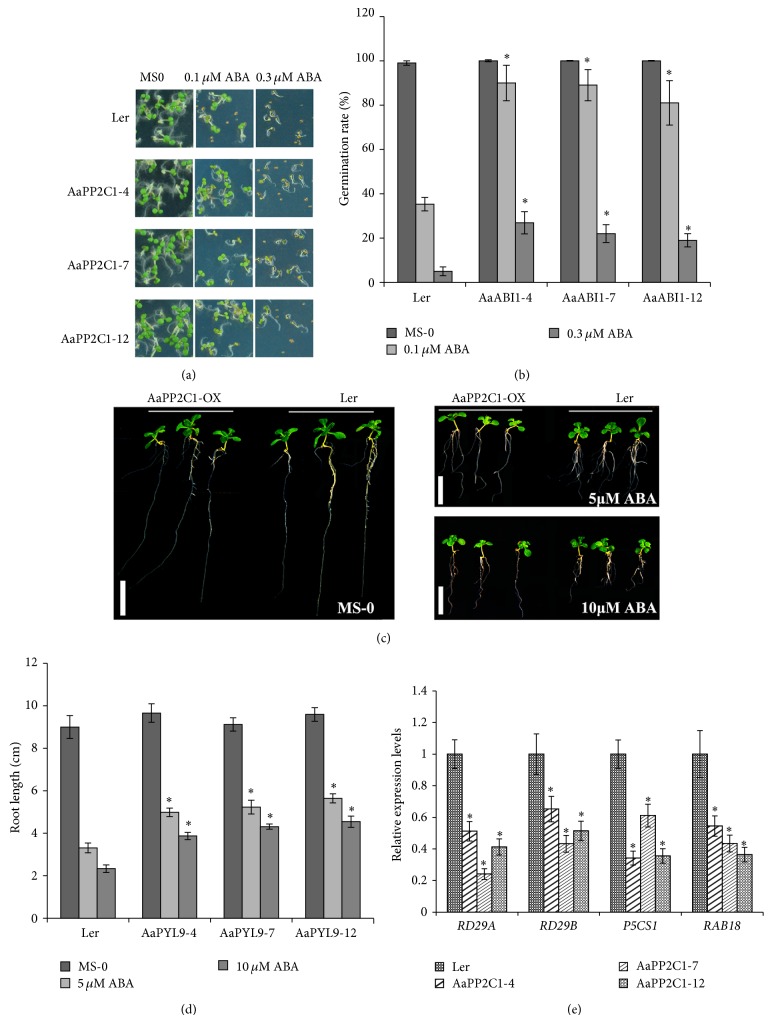
Comparative analysis of seed germination and root length between* AaPP2C1*-OX and wild type* Arabidopsis*. (a) Seed germination rate of* AaPP2C1*-OX* Arabidopsis* higher than wild type. (b) Statistics of seed germination rate. (c) The root length of* AaPP2C1*-OX* Arabidopsis* longer than wild type in the presence of the indicated ABA concentrations is shown. (d) Statistics of root length. (e) Reduced expression of ABA-inducible genes in* AaPP2C1* overexpression* Arabidopsis*. The mRNA levels of the indicated genes were determined by Q-PCR analysis using total RNAs isolated from 10 *μ*M ABA-treated plants for 3 h. SD (standard deviation) indicates three independent tests. ^*^ *P* < 0.01, *t*-test.

The phenotype of* AaPYL9*-overexpression in* Arabidopsis* was previously reported to be ABA hypersensitivity [26]. As AaPP2C1 interaction with AaPYL9, we performed constitutively expression* AaPP2C1* in* Arabidopsis*. As shown in Figure 4(a), the AaPP2C1-overexpression lines exhibited higher frequency of seedlings with green cotyledons under 0.1 *μ*M ABA or 0.3 *μ*M ABA treatment compared with wild type. The percentage of seeds that germinated in the presence of indicated concentrations of ABA was also determined (Figure 4(b)). These assays reveal a reduced sensitivity to ABA of* AaPP2C1* overexpression lines. Moreover, this phenotype was apparent in root length assay, when seedlings were grown in MS medium with 5 *μ*M ABA or 10 *μ*M ABA for 7 d. The roots of all the AaPP2C1-overexpression lines showed 50–95% longer than wild type root (Figures 4(c) and 4(d)). These results suggest that AaPP2C1 is a negative regulator of ABA signaling during seed germination and root elongation.

Further evidence of the function of AaPP2C1 in ABA signaling was obtained through analyzing the expression of the ABA-responsive* RD29A*,* RD29B*,* P5CS1,* and* RAB18* genes in the wild type and the* AaPP2C1*-overexpression lines by quantitative real-time PCR (qRT-PCR). Upon ABA treatment, the expression levels of* RD29A*,* RD29B*,* P5CS1*, and* RAB18* genes were significantly reduced in AaPP2C1 overexpression lines compared with wild type (Figure 4(e)).

## 4. Conclusions

In this study, we functionally characterized AaPP2C1, a clade A PP2C, in* Artemisia annua* L. Previously, we reported an ABA receptor in* Artemisia* which act as a positive regulator in ABA signaling and play an important role in ABA signaling regulating artemisinin content. Yeast two-hybrid assay and BiFC analysis revealed a strong interaction between AaPYL9 and AaPP2C1. Both the functional PP2C catalytic domain and the third phosphorylation site-like motif of AaPP2C1 appeared to be required for the interaction (Figure 3). Overexpression of* AaPP2C1* in* Arabidopsis* reduced ABA sensitively in seed germination and root elongation revealed that AaPP2C1 is a negative regulator of ABA signaling (Figure 4). Taken together, our data suggests that AaPP2C1 is a functional PP2C in* Artemisia*, showing that the core regulatory mechanism of early ABA signaling in* Artemisia* is conserved with* Arabidopsis*. This is particularly important for investigating the mechanism ABA signaling regulation secondary metabolism; AaPYL9 especially has been reported to be involved in regulating artemisinin biosynthesis.

## Supplementary Material

Supplementary Figure 1: Phylogenetic analysis of AaPP2C1 and sixty-eight Arabidopsis type-2C protein phosphatases (PP2Cs).
Supplementary Table 1: Real time-PCR Primers used in this study.
