# Corrigendum to “Type 2C Phosphatase 1 of* Artemisia annua* L. Is a Negative Regulator of ABA Signaling”

**DOI:** 10.1155/2016/3565916

**Published:** 2016-08-17

**Authors:** Fangyuan Zhang, Xueqing Fu, Zongyou Lv, Qian Shen, Tingxian Yan, Weimin Jiang, Guofeng Wang, Xiaofen Sun, Kexuan Tang

**Affiliations:** Plant Biotechnology Research Center, Fudan-SJTU-Nottingham Plant Biotechnology R&D Center, School of Agriculture and Biology, Shanghai Jiao Tong University, 800 Dongchuan Road, Shanghai 200240, China

In the article titled “Type 2C Phosphatase 1 of* Artemisia annua* L. Is a Negative Regulator of ABA Signaling” [1], the name of the sixth author was incorrectly written as Weiming Jiang while the correct name is Weimin Jiang. The correct authors' list is shown above.
