# Supplementary figures and images for: Type 2C Phosphatase 1 of Artemisia annua L. Is a Negative Regulator of ABA Signaling
Source: Biomed Res Int. 2014 Oct 28;2014:521794. doi: 10.1155/2014/521794 (PMC4228716; doi:10.1155/2014/521794)

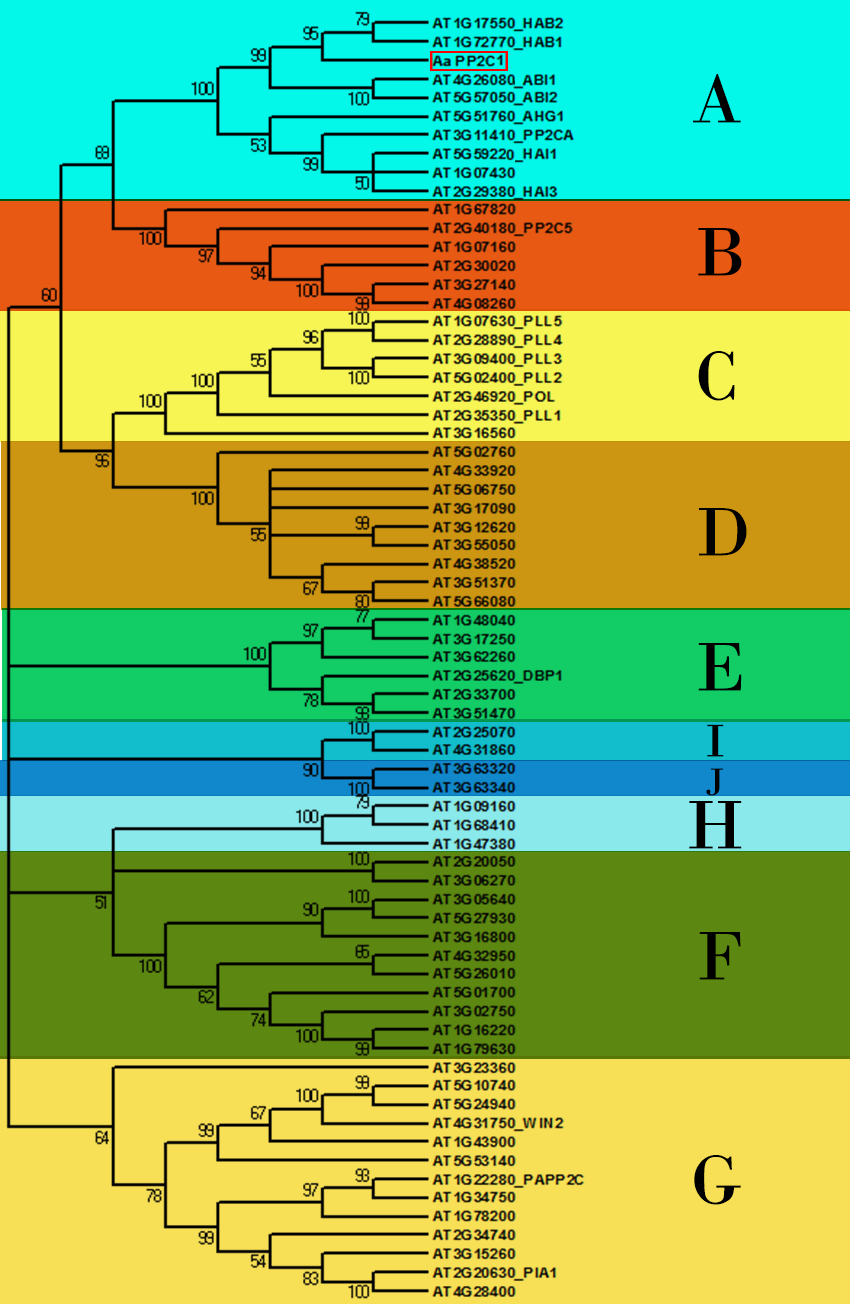

Supplement: Supplementary file 1 — Supplementary Figure 1: Phylogenetic analysis of AaPP2C1 and sixty-eight Arabidopsis type-2C protein phosphatases (PP2Cs). Supplementary Table 1: Real time-PCR Primers used in this study. [file 521794.f1.docx]
