# Supplementary material for: Type 2C Phosphatase 1 of Artemisia annua L. Is a Negative Regulator of ABA Signaling
Source: Biomed Res Int. 2014 Oct 28;2014:521794. doi: 10.1155/2014/521794 (PMC4228716; doi:10.1155/2014/521794)
Supplement: Supplementary file 2 [file 521794.f2.docx]

**Supplemental Table 1: Real time-PCR Primers used in this study**

| Oligonucleotide (5’-3’) | Primer name |
| --- | --- |
| GTTACTGATCCCACCAAAGAAGA | **rd29a-qF** |
| GGAGACTCATCAGTCACTTCCA | **rd29a-qR** |
| GGCTTGGGAGGAATGCTT | **RAB18-qF** |
| TTGATCTTTTGTGTTATTCCCTTCT | **RAB18-qR** |
| GGCGGGCAAAGCGAG | **RD29B-qF** |
| TGCCCGTAAGCAGTAACAGATC | **RD29B-qR** |
| TGTGTGTTTGTGTATTTGGTTGAGAC | **P5CS1-qF** |
| TGAGTACTAAGCAGAGAGGAAACAAAA | **P5CS1-qR** |
| AGGTAGATTGTAAAGAACCCTT | **PP2C-qF** |
| AAGTAACCGAAACCGAAG | **PP2C-qR** |
| CCAGGCTGTTCAGTCTCTGTAT | **AaACTIN-qF** |
| CGCTCGGTAAGGATCTTCATCA | **AaACTIN-qR** |
| tcagcactttccagcagatg | **AtACTIN-qF** |
| ctgtggacaatgcctggac | **AtACTIN-qR** |
